# Haploinsufficiency of EXT1 and Heparan Sulphate Deficiency Associated with Hereditary Multiple Exostoses in a Pakistani Family

**DOI:** 10.3390/medicina59010100

**Published:** 2022-12-31

**Authors:** Muhammad Ajmal, Hafsah Muhammad, Muhammad Nasir, Muhammad Shoaib, Salman Akbar Malik, Irfan Ullah

**Affiliations:** 1 Institute of Biomedical and Genetic Engineering, 24-Mauve area, G-9/1, Islamabad 44000, Pakistan; 2 Department of Molecular Biology and Genetics, Institute of Basic Medical Science, Faculty of Basic Medical Sciences, Khyber Medical University, Phase V Hayatabad, Peshawar 25000, Pakistan; 3 KRL General Hospital, Orthopaedic Department 24-Mauve area, G-9/1 Islamabad 44000, Pakistan; 4 Department of Biochemistry, Quaid-i-Azam University, Islamabad 44000, Pakistan; 5 Department of Life Sciences, School of Science, University of Management and Technology (UMT), Lahore 54770, Pakistan

**Keywords:** hereditary multiple exostoses, *EXT1*, *EXT2*, heparan-sulfate, haploinsufficiency

## Abstract

*Background and Objectives*: Hereditary multiple exostoses (HME) is a disease characterized by cartilage-capped bony protuberances at the site of growth plates of long bones. Functional mutations in the exostosin genes (*EXT1* and *EXT2*) are reported to affect the hedgehog signalling pathways leading to multiple enchondromatosis. However, the exact role of each EXT protein in the regulation of heparan sulphate (HS) chain elongation is still an enigma. In this study, a Pakistani family with HME is investigated to find out the genetic basis of the disease. *Materials and Methods*: Genotyping of eight members of the family by amplifying microsatellite markers, tightly linked to the *EXT1* and *EXT2* genes. *Results*: The study revealed linkage of the HME family to the *EXT1* locus 8q24.1. Sanger sequencing identified a heterozygous deletion (*c.247Cdel*) in exon 1 of *EXT1*, segregating with the disease phenotype in the family. In silico analysis predicted a shift in the frame causing an early stop codon (p.R83GfsX52). The predicted dwarf protein constituting 134 amino acids was functionally aberrant with a complete loss of the catalytic domain at the C-terminus. Interestingly, an alternative open reading frame 3 (ORF3) caused by the frame shift is predicted to encode a protein sequence, identical to the wild type and containing the catalytic domain, but lacking the first 100 amino acids of the wild-type EXT1 protein. *Conclusion*: Consequently, haploinsufficiency could be the cause of HME in the investigated family as the mutated copy of *EXT1* is ineffective for *EXT-1/2* complex formation. The predicted ORF3 protein could be of great significance in understanding several aspects of HME pathogenesis.

## 1. Introduction

Hereditary (HME) or hereditary multiple osteochondroma is a genetically heterogeneous bone condition inherited as an autosomal dominant trait [1]. It is characterized by cartilage-capped bony outgrowths on the external surface of long bones leading to shortening and bowing of the forearm and reduced skeletal growth [2]. The prevalence of the disease is estimated at 1:50,000, and in general, males tend to be more severely affected than females [3].

Genetically, HME is caused by heterozygous mutations of *EXT1* or *EXT2* genes, which encode exostosin-1 and exostosin-2 glycosyltransferases, respectively [4]. Both EXT1 and EXT2 proteins are predominantly expressed in the endoplasmic reticulum (ER) where they form the hetero-oligomeric EXT-1/2 complex before moving to the Golgi apparatus. In the Golgi apparatus, the hetero-oligomeric EXT-1/2 complex plays a significant role in HS biosynthesis. Initiation of HS biosynthesis starts with the formation of tetrasaccharide unit (xylose–galactose–galactose–glucuronic acid (GlcA)) that covalently links to a serine residue on the HS proteoglycan (HSPG) core protein. More specifically, the exostosin-like 2 protein (EXTL2) initiates HS chain formation by transferring the first N-acetylglucosamine (GLcNAc) residue to the tetrasaccharide linker sequence, thereby providing the substrate for HS polymerization by EXT1/2 complex. The EXT-1/2 complex polymerizes HS biosynthesis by adding repeating disaccharide units (GlcA and GlcNAc) to the growing polymer [5].

HS is a ubiquitous component of the extracellular matrix and interacts with a number of specific signalling proteins including Indian hedgehog (IHH), bone morphogenetic proteins (BMPs), fibroblast growth factor (FGF), Wnt growth factors, extra cellular enzymes, and cytokines. The interaction regulates the distribution, respective interactions, function, and bioactivity of signal molecules on target cells [6]. During perichondrial ossification, HS interacts with IHH and facilitates its binding to receptor molecule patched protein (Ptc). IHH–Ptc interaction in turn induces the expression of parathyroid hormone-related peptide (PTHrP) in the perichondrial space. IHH and PTHrP are the key players in controlling the chondrocyte proliferation, differentiation, and maturation of skeletal development through a negative feedback mechanism [7,8,9]. Loss of this negative feedback mechanism due to impaired HS formation or malfunction of any of the above-mentioned pathways may lead to uncontrolled bone growth or exostosis. Impaired HS formation or its deficiency causes an increased diffusion of signalling proteins that otherwise remain restricted by binding to HS, and hence might initiate exostosis formation [10,11,12].

Although the knowledge on HS synthesizing and modifying proteins in skeletal development is still quite limited, their significance is evident by the fact that several skeletal phenotypes are caused by genetic mutations in *EXT1* and *EXT2* genes [13]. Most of the mutations (60–70%) are found in *EXT1* comprising mainly nonsense, frame shift, or splice-site mutations that result in premature termination and nearly complete loss of EXT1 protein function. Therefore, severe HME phenotypes are mainly reported as being associated with *EXT1* mutations rather than *EXT2* [14].

Here we report a Pakistani family with HME and identify a heterozygous deletion mutation in HS-synthesizing EXT1 protein which might have led to its haploinsufficiency and ultimately disease phenotypes.

## 2. Material and Methods

### 2.1. Subjects

**Figure 1:**
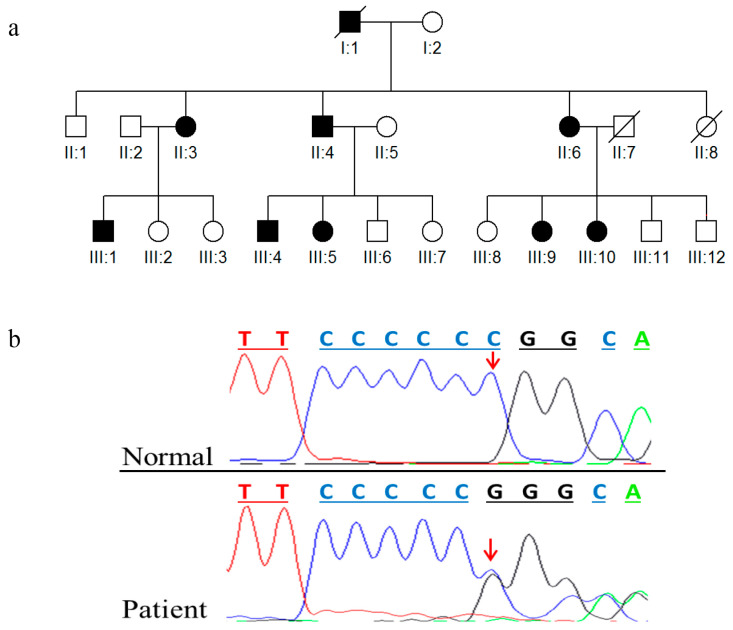
Sequencing analysis of exon 1 of *EXT1* gene. (**a**) Pedigree of affected HME family. (**b**) Partial electropherograms of exon 1 of EXT1 gene; arrows indicate the point of deletion of cytosine at nucleotide position 247 (c.247delC). Phenotypically healthy individuals exhibit homozygous status [C/C], while affected individuals are with heterozygous status [C/G].

In this study, we recruited a multigenerational Pakistani family diagnosed with HME (Figure 1a). Affected individuals II:4, II:6, III:4, III:5, III:9 with age ranges from 20–65 years and healthy individuals II:5, III:6, III:8 participated in this study. Severity of the disease phenotypes was moderate in (II:6 & III:9) and severe in (II:4, III:4 & III:5) and with varying sites and sizes of osteoenchondromas in affected subjects. The disease symptoms at birth became increasingly prominent with the passage of time, until the cessation of developmental growth process. Subjects consented to the study. Blood samples were drawn from all subjects. A panel of 100 ethnically matched unrelated healthy individuals was also enrolled to determine the disease association of identified variation and its frequency in the normal population.

The study was approved by AS&RB Quaid-i-Azam University, Islamabad, Pakistan as well as its institutional ethics committee (Ethical Committee, IB&GE, Islamabad, Pakistan) and was in concordance with the Helsinki declaration.

### 2.2. Genotyping and Mutation Analysis

Linkage analysis approach was used to localize the disease gene. The markers used to amplify the microsatellite loci within or adjacent to the candidate genes (*EXT1* and *EXT2*) for HME were D8S556 (120.4cM), D8S1132 (123.8cM), D8S592 (128.2cM), D8S199 (130.5cM), and D8S514 (134.2cM) for locus 8q24.1 and D11S1392 (35.3cM), D11S935 (38.3cM), D11S905 (40.6cM), D11S1361 (45.9cM), and D11S1313 (58cM) for locus 11p11.2, respectively. The disease was mapped to the locus 8q24.1 harbouring the *EXT1* gene. To identify the underlying pathogenic mutation, 11 sets of intronic primers were designed to amplify the coding sequences and exon–intron boundaries of *EXT1* gene. Purified PCR products were sequenced directly by using BigDye^®^Terminator v3.1 cycle sequencing kit on an ABI3130 genetic analyzer (Applied Biosystems, Waltham, MA, USA). Potential disease-associated mutation was confirmed through bidirectional sequencing of all affected, phenotypically normal individuals and 100 control samples with a similar ethnic background. Reference sequence for *EXT1* gene (NG_007455.2) was retrieved from NCBI.

### 2.3. In Silico Analysis

We determined alternative open reading frames (ORFs) in *EXT1*_c.247delC using ExPASy Translate tool [15] and predicted functional domains on selected ORFs using Interproscan [16]. We used I-TASSER [17] to predicted protein 3D structures and superpositioned the resulting models in pymol [18]. Root-mean-square deviations (RMSDs) of predicted structures were calculated using SuperPose [19].

## 3. Results

### 3.1. HME Clinical Heterogeneity within a Pakistani Family

**Figure 2:**
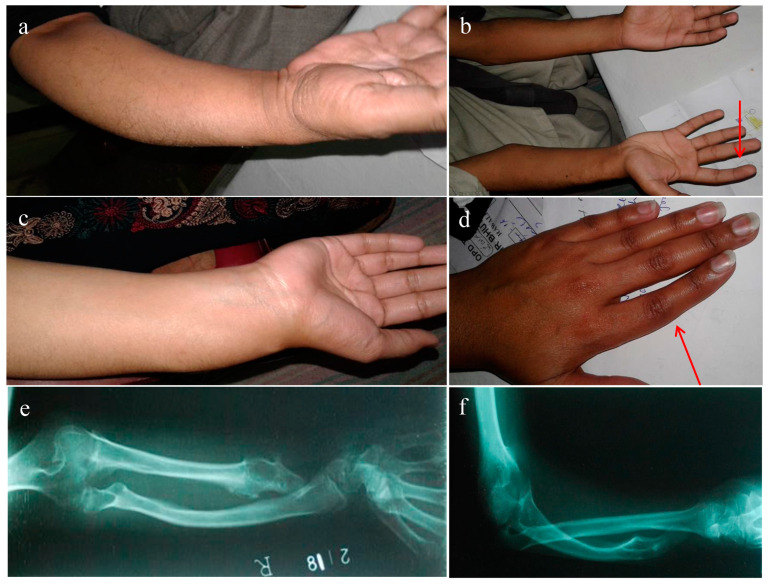
Clinical manifestation of HME family. (**a**) Patient II:4 is representing varus deformities and radial bowing due to ulnar shortening. (**b**) Patient III:4, demonstrating limbs malalignment, limbs length discrepancy along with cubitus valgus deformity at elbows with limited joints movement and clinodactyly. (**c**) Patient III:5 with varus deformities of the elbow. (**d**) Patient III:5 shows clinodactyly as pointed by arrow. (**e**,**f**) Radiological examination of patient III:4 showing ulnar shortening, radial bowing and dislocated radioulnar joints respectively.

The most common phenotypic findings in the majority of our cases were radial bowing secondary to ulnar shortening and dislocated radio-ulnar joints (Figure 2a–c). The limb misalignment and limb length discrepancy were noticed in patient III:4 (Figure 2b). The movement was observed to be limited at joints where the lesions were present. An important clinical feature of HME is the valgus or varus deformities of knees and elbows. However, in the current family, only the elbow deformities were observed, with one patient III:4 possessing a cubitus valgus deformity (Figure 2b), while the rest of the patients showed a varus of the elbows, in patients II:4 & III:5. Deformity of the knee joints was not found in our patients. The ulnar deviation of the index finger of hands (clinodactyly) was obvious in two of our patients (III:4 and III:5), most likely due to rotational deformity of the inter-phalangeal joints of hands (Figure 2b,d). Clinodactly was not observed in the remaining affected family members. The radiological findings of patients III:4 revealed an ulnar shortening (Figure 2e) and dislocated radio-humeral joints (Figure 2f). Moreover, the clinical examination of affected female individuals in the family identified a higher number of exostoses on their bodies than the male patients. Severely affected female subjects (II:6 and III:9) opted out of being photographed and only the less affected female subject (III:5) allowed photography of her forearms (Figure 2c,d). The affected subjects endured constant pain because of their limb deformities. However, no axial skeletal shortening was observed in this family.

### 3.2. HME Patients Carry Frameshift Mutation in EXT1 Gene

Genotyping linked the disease to locus 8q24.1 that harbours the *EXT1* gene, previously associated with HME. The markers were highly polymorphic; therefore, the heterozygous status in all affected individuals confirmed that the disease is following an autosomal dominant mode of inheritance in the family.

Sanger sequencing identified a heterozygous deletion of cytosine at position 247 (*c.247Cdel*) in exon 1 of the *EXT1* gene (Figure 1b). Cytosine deletion not only causes a substitution of arginine with glycine at amino acid position 83 but also a shift in the reading frame of EXT1. All affected individuals in the family were found to be heterozygous for this change, whereas phenotypically healthy subjects carried the wild type genotype. The identified variation was not found in a panel of 100 ethnically matched control samples.

### 3.3. Frameshift Mutation Leads to Premature Stop Codon in EXT1

**Figure 3:**
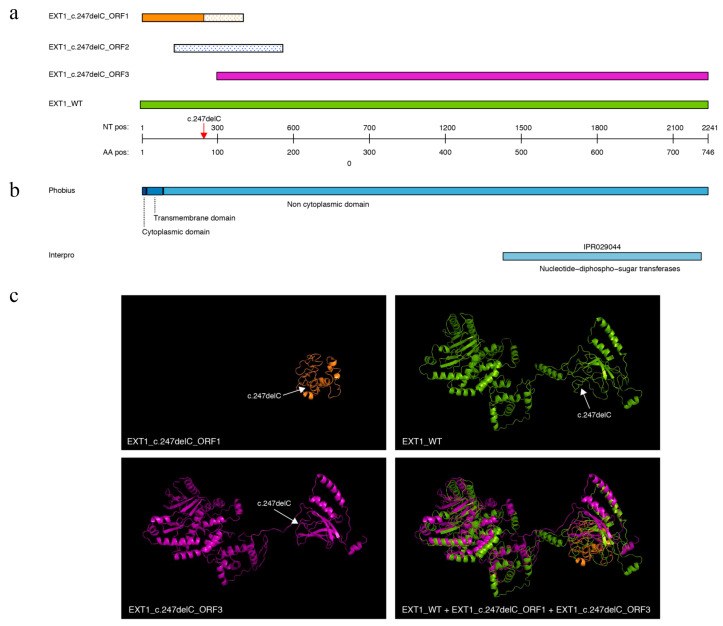
Predicted tertiary structures and protein domains of predicted open reading frames (ORFs) in *EXT1*_c.247delC. (**a**) Location of ORFs on *EXT1* gene sequence. Solid colours indicate that amino acid sequence of ORFs in *EXT1*_c.247delC (orange, blue, magenta) are identical to wild type (green). (**b**) Predicted cytoplasmic location and functional domain on wild type amino acid sequence. (**c**) Predicted 3D structures of EXT1 wild type (green) and two ORFs of *EXT1*_c.247delC (orange, magenta).

Due to a frame shift caused by the c.247delC mutation, the *EXT1* gene has an early stop codon on frame 1 at nucleotide position 403. Thus, the length of the translated protein sequence is only 134 amino acids instead of the normal 746 amino acids (Figure 3a, orange bar). Like the wild type, the shorter protein seems to be membrane bound as it contains a predicted transmembrane domain (Figure 3b). Considering alternative open reading frames (ORFs), we found two ORFs in the mutant gene that are longer than the interrupted ORF on frame 1. One alternative ORF on frame 2 (Figure 3a, blue dotted bar) is predicted to be 142 amino acids long and has no similarity to the wild type protein. The third ORF is on frame 3 (Figure 3a, magenta bar) and translates into an amino acid sequence of 647 residues, which is identical to the wild type sequence between residue position 100 and the C terminus. Therefore, it also encompasses the nucleotide-diphospho-sugar transferases domain (Figure 3b). Tertiary structure predictions also revealed the structural similarity of the alternative ORF on frame 3 to the wild type structure (RMSD local: 16.82, RMSD global: 93.33) (Figure 3c).

## 4. Discussion

Heparan sulphate (HS) biosynthesis and subsequent glycosylation, polymerization, sulphation, and epimerization are vital for skeletal development [20]. HS biosynthesis depends on proper functioning of the EXT-1/2 complex regulated by *EXT1* and *EXT2* genes. In the past, several studies identified the functional variants of EXT1 and EXT2 as associated with impaired EXT-1/2 complex formation and HS biosynthesis, leading to abnormal endochondral ossification or exostoses formation [21].

The present study identified an already reported heterozygous deletion mutation in exon 1 of the *EXT1* gene (c.247delC; pA83G). This frame shift variation resulted in an early stop codon 52 amino acids downstream of the point of deletion. The resulting protein is predicted to consist of only 134 amino acids, constituting a loss of 612 amino acids and therefore a considerable part of the EXT1 protein. The presence of the heterozygous status of the mutation in all affected individuals but not in phenotypically normal subjects as well as 100 ethnically matched healthy controls supports its association to HME in this Pakistani family.

Structurally, the EXT1 protein is composed of a transmembrane domain (16–20 amino acids long) followed by a stem region (35–62 amino acids long) at the N-terminal end, an exostosin interaction domain (110–396 amino acids) in the centre and a catalytic domain/nucleotide-diphospho-sugar transferase domain (480–729 amino acids) at the C-terminal [1,22]. In the present study, identified deletion variation (pA83GfsX52) is lying towards the N-terminal of EXT1 protein possibly in the stem region, leading to a frameshift and ultimately resulting in a shorter protein of 134 amino acids. This shorter protein product encoded by ORF-1 is predicted to possess a transmembrane domain while lacking two significant domains, the central exostosin domain and the catalytic glycosyltransferase domain in the C-terminus.

The exact role of EXT proteins in the regulation of HS chain elongation is still an enigma. In light of prior studies, the transmembrane region of EXT1 is suggested to remain membrane bound while the catalytic region exclusively participates in EXT-1/2 complex formation and HS biosynthesis. Some EXT1 transmembrane domain deficient yeast models with functional catalytic domains have demonstrated the formation of the EXT-1/2 complex and normal HS biosynthesis [23,24]. The predicted absence of the catalytic region in the *EXT1* deletion variant may prevent the formation of the EXT-1/2 complex and avert HS synthesis. Therefore, haploinsufficiency for EXT1 might be responsible for the HME phenotype. Consequently, impaired and/or decreased HS chain synthesis will affect the binding potential of the HS chain to its functional partner molecules. The abrogated HS synthesis affecting downstream cellular signalling pathways (Ihh, BMPs, FGF, Wnt) might lead to the development of HME phenotypes [25,26]. It might also be possible that the mutated protein exerts a dominant negative effect on the activity of the wild type protein [27]. However, the type and site of the mutation, along with the residual protein activity, could be determining factors for the dominant negative effect. Another hypothesis involves the activation of nonsense-mediated mRNA decay (NMD) to eliminate the EXT1 gene transcripts carrying premature termination codon [28], resulting in a complete loss of one copy of EXT1. Although information about HME is rapidly emerging, the pathogenesis remains a mystery. Based on the findings of this study, we conclude that that haploinsufficiency is most likely the underlying cause of the disease phenotypes in this family as one copy of EXT1 is lacking the catalytic domain and not contributing to the formation of EXT-1/2 complex.

Alternative proteins constitute up to 55% of the total proteome [29,30]. It is remarkable that despite the frame shift in EXT1 an alternative open reading frame translates into a 647 amino acid sequence, which is identical to the C-terminal end of the wildtype EXT1. If this alternative ORF was translated, the glycosyltransferase activity might still be possible due to the presence of the nucleotide-diphospho-sugar transferase domain. However, it is questionable if a stable EXT1-1/2 complex formation would still be possible given the missing N-terminus and transmembrane domain. In any case, to show whether the alternative ORF is actually transcribed and translated, additional investigations, including transcriptomics and proteomics experiments, will be necessary.

## 5. Conclusions

In short, the presence of clinical heterogeneity within our HME family could be of interest for clinicians in expanding their understanding on disease pathophysiology that might be helpful in personalized medicine. The frame shift mutation and resultant impaired EXT1 protein-lacking catalytic domain might be the possible cause of HME in our Pakistani family. However, in silico analysis in the study predicts an alternative ORF translating the entire catalytic domain identical to the wild type, required for HS chain elongation. Future studies based on our genetic findings and in silico predictions could help to get a deeper discernment of the aspects of EXT-1/2 complex formation, HS biosynthesis, and HME pathogenesis. Therefore, this study might also be helpful in prenatal genetic screenings and early diagnosis of HME in the Pakistani population.

## Data Availability

The data that support the findings of this study are with corresponding authors, it’s not publicly available due to privacy of participant patients. If needed contact with corresponding authors.
